# Automated assessment of simulated laparoscopic surgical skill performance using deep learning

**DOI:** 10.1038/s41598-025-96336-5

**Published:** 2025-04-19

**Authors:** David Power, Cathy Burke, Michael G. Madden, Ihsan Ullah

**Affiliations:** 1 ASSERT Centre, College of Medicine and Health, University College Cork, Cork, Ireland; 2 Cork University Maternity Hospital, Cork, Ireland; 3 School of Computer Science, University of Galway, Galway, Ireland; 4 Insight Research Ireland Centre for Data Analytics and Data Science Institute, University of Galway, Galway, Ireland

**Keywords:** Laparoscopic Surgery, Automated Assessment, Deep Learning, 3DCNN, Health care, Medical research, Mathematics and computing

## Abstract

Artificial intelligence (AI) has the potential to improve healthcare and patient safety and is currently being adopted across various fields of medicine and healthcare. AI and in particular computer vision (CV) are well suited to the analysis of minimally invasive surgical simulation videos for training and performance improvement. CV techniques have rapidly improved in recent years from accurately recognizing objects, instruments, and gestures to phases of surgery and more recently to remembering past surgical steps. Lack of labeled data is a particular problem in surgery considering its complexity, as human annotation and manual assessment are both expensive in time and cost, and in most cases rely on direct intervention of clinical expertise. In this study, we introduce a newly collected simulated Laparoscopic Surgical Performance Dataset (LSPD) specifically designed to address these challenges. Unlike existing datasets that focus on instrument tracking or anatomical structure recognition, the LSPD is tailored for evaluating simulated laparoscopic surgical skill performance at various expertise levels. We provide detailed statistical analyses to identify and compare poorly performed and well-executed operations across different skill levels (novice, trainee, expert) for three specific skills: *stack, bands, and tower*. We employ a 3-dimensional convolutional neural network (3DCNN) with a weakly-supervised approach to classify the experience levels of surgeons. Our results show that the 3DCNN effectively distinguishes between novices, trainees, and experts, achieving an F1 score of 0.91 and an AUC of 0.92. This study highlights the value of the LSPD dataset and demonstrates the potential of leveraging 3DCNN-based and weakly-supervised approaches to automate the evaluation of surgical performance, reducing reliance on manual expert annotation and assessments. These advancements contribute to improving surgical training and performance analysis.

## Introduction

Minimally invasive surgery (MIS) offers numerous patient and organizational advantages, such as reduced pain post-operatively, lower incidence of operative and post-operative major complications, less scarring, smaller incisions, lower immune system stress, and faster recovery times^1–4^. However, it is not without risk; a study in the UK and Ireland found 47% of surgeons reported performing an error during MIS in the preceding 12 months, while 75% knew of a surgical colleague who had^5^. There are similar findings in the US, where 8.9% of surgeons reported making a major error in the previous 3 months^4^. Surgical complications occur most frequently during the first 10 procedures that trainee MIS surgeons perform^5^. For example, bile duct injuries from MIS, which are associated with a threefold increase in mortality in one year, cost over $1 billion in the US alone, with increased hospital stays and litigation. The rate of bile duct injuries has not improved over the last three decades, and the rate of incidence is three times higher in MIS compared to open surgery^6^. Furthermore, surgical technical errors are a leading cause of preventable patient harm, with a 2016 review finding that if medical errors were classified as a disease in the US, it would be the third leading cause of death^7^.

Mastering MIS skills is a significant undertaking, and requires skill acquisition well beyond those of open surgery^3^. Unlike open surgery, MIS lacks direct tactile tissue palpation, degrading tactile feedback as the surgeon is using instruments through trocar ports or robot manipulators. There is an inversion of the perceptual-motor correlation, due to the fulcrum effect posed by the patient’s abdominal wall, as the surgeon moves their hand in one direction the working end of the instrument moves in the opposite direction. There is also a loss of binocularity, as the surgeon must form impressions of 3D anatomical structures while tracking instruments, devices, and other hidden structures from available 2D images from the monitor^3,4^. These are difficult, time-consuming skills to master, and are prone to error, especially for trainee surgeons^6^.

However, a significant portion of teaching and learning occurs in the operating theatre, often following on from a period of simulation-based training using VR simulators or surgical box trainers where skills such as suturing, knot tying, needle passing, and instrument handling are honed^8^. The acquisition of high-quality surgical skills is a time-intensive process for experts with regard to both supervision and evaluation throughout the entire training pathway. Tools such as the objective structured assessment of technical skills (OSATS) were developed to reduce subjectivity, but it remains a time-consuming manual assessment^9^. Trainees must master basic skills such as instrument handling and tissue manipulation, then demonstrate competence in suturing and knot-tying before moving on to more complex tasks^6,10^. The use of video can be a powerful assessment tool for both discrete surgical skills and an entire surgical procedure. In fact, a positive correlation has been demonstrated between video-based surgical skill assessment and postoperative patient outcomes^11,12^. The automation of skills assessment potentially offers massive benefits in surgery, through computer vision techniques.

The rest of the paper is organized as follows. Section 1 will give background, related works, and their limitations. Section 2 will explain the methodology, and describe LSPD data acquisition, augmentation, and the proposed DL model. Experiments and their analysis are given in Section 3, the results are presented in section 4. Finally, Section 5 will conclude the paper.

## Background and related works

**Fig. 1:**
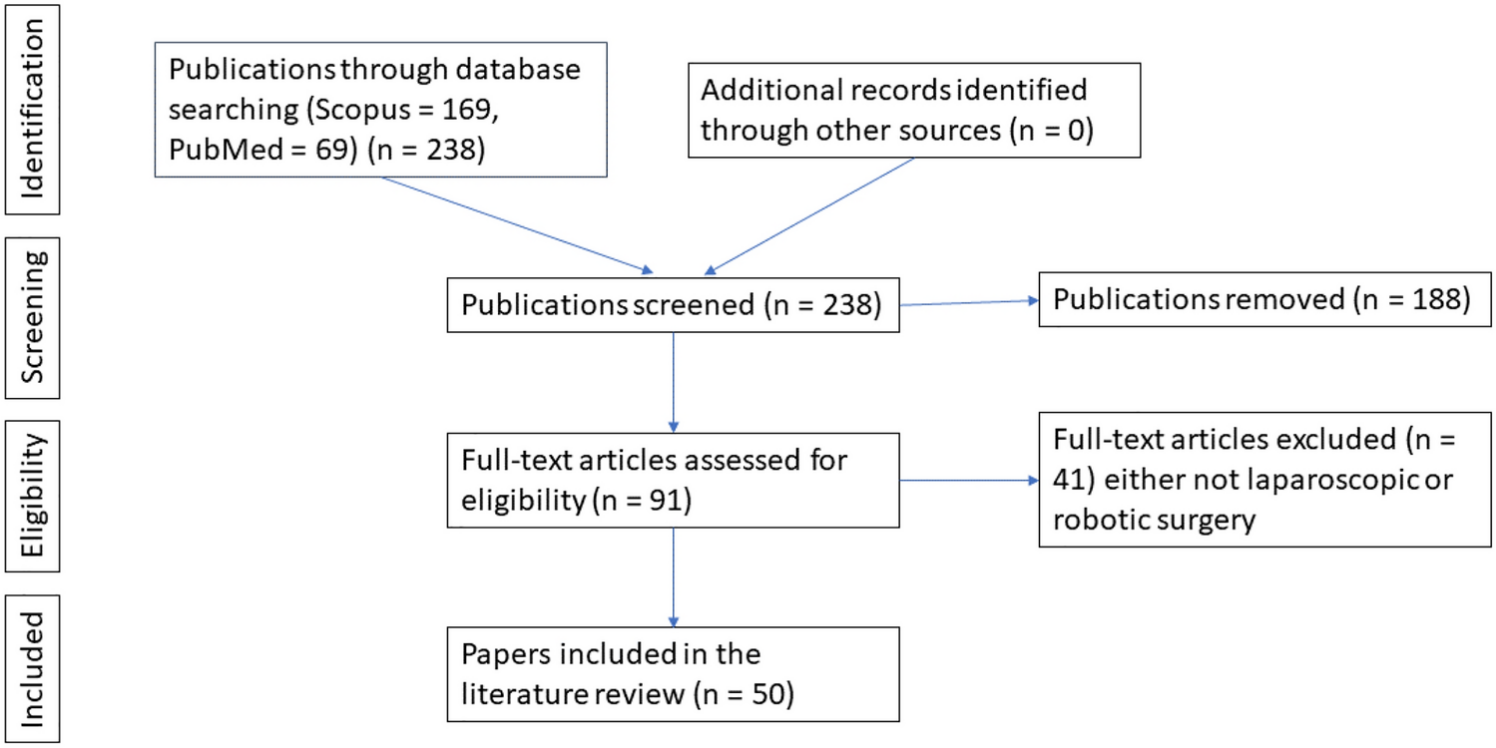
Literature selection process.

**Literature review:** A literature review was undertaken to gain an understanding of the current use and state-of-the-art CV for training and assessment in MIS. The search terms used in Google Scholar were initially ‘Computer Vision’ and ‘Laparoscopic Surgery’ which yielded results across a spectrum of journals from surgery and computer science. Further terms such as ‘Neural Networks’, ‘Artificial Intelligence’, and ‘Machine Learning’ were used with combinations of ‘Laparoscopy’, ‘Laparoscopic’, and ‘Minimally Invasive Surgery’. The search was also widened to include ‘Robotic Surgery’ and ‘Robot Assisted Surgery’, as this is a form of computer and robotic-assisted laparoscopic surgery. On each iteration, the journals were examined and fell across several domains, namely surgery, computer vision, and to some extent biomedical engineering. The search continued using Scimago to check both the H-index and Scimago Journal Rank (SJR) of each of the journals identified. Scimago was also used to identify other journals of relevance in similar categories not yet identified. Mostly the journals identified had high SJR scores of Q1. There were several with a lower score of Q2 and one with a score of Q3, however, several papers were identified in these journals that may be of use for the literature review. Once the SJR score was identified for specific journals, searches were widened further to include Scopus (n=169) and PubMed (n=69) for medical/surgical journals (Fig. 1). Further searches were carried out on IEEE for conference papers. Ninety-one papers were then selected from the first sweep of reading the title, and abstract. The abstract, conclusion, and main findings were then scrutinized to determine which papers to retain for the literature review based on relevance, reducing the final number in the literature review to fifty.

The literature reveals that AI and CV demonstrates considerable promise in the surgical field, particularly in the analysis of high-definition surgical video, which can contain up to 25 times the volume of data found in a high-resolution CT scan^13^. The use of AI and CV techniques in surgery has primarily focused on object detection and gesture recognition, with the aim of augmenting the process of surgical training and performance evaluation. Despite their potential, the lack of suitable, high-quality annotated data for training machine learning models remains a significant challenge, as manual annotation requires expert involvement, which is both time-consuming and costly. This bottleneck is especially problematic in complex surgical tasks, where expert annotation is necessary for accurate skill classification.

Several efforts have been made to mitigate this issue. For instance, crowdsourcing of annotations and the use of semi-supervised and unsupervised machine learning methods have been explored^14^. However, expert knowledge remains central for accurately assessing surgical performance, and these methods are not always sufficient for nuanced skill classification, particularly in laparoscopic surgery.

Most open-source laparoscopic datasets, such as Cholec80, SurgAI, Heidelberg Colorectal, LapGyn4, and Dresden Surgical Anatomy, are designed for detecting surgical phases, tracking anatomical structures, or instrument identification^15–19^. These datasets, while valuable for instrument tracking and phase recognition, do not lend themselves easily to classifying laparoscopic surgical performance skill levels (i.e., novice, trainee, expert) in the simulated environment. Similarly, while the JIGSAWS dataset was designed for surgical robotic gesture and skill assessment, it does not provide the necessary annotations or features to evaluate laparoscopic surgical skills in a simulated setting^20^. As such, there is still a significant need for datasets that can easily lend themselves to the task of automating the classification of surgical skill performance levels.

Many previous studies in the past have described successful systems for assessing surgical training involving tracking instruments, navigation, gesture recognition, and real-time knowledge of their pose with respect to underlying anatomy and tissue. These systems have employed techniques such as electromagnetic tracking, optical tracking, and robot kinematics^21–23^. In the early 2000s, CV approaches for instrument tracking were introduced, using pre-processing, feature extraction, and filtration methods^24^. Other techniques like Continuous Adaptive Mean Shift (CamShift) have aimed to replicate the actions of surgeons during simulator-based training^19^. A common approach is tracking, which has been framed as an object detection problem in which image features are used to estimate the position and orientation of instruments. Instrument tracking and gesture recognition in laparoscopic surgery have generally relied on traditional CV techniques, including the use of colour and gradient features to detect instruments and surgical gestures^26–28^. While reasonably effective, these techniques suffer from limitations due to lighting reflections and occlusions during surgery, which can distort the appearance of instruments and interfere with tracking accuracy^1,26^. As a result, some researchers have turned to semantics-based approaches, which use classification algorithms to identify surgical objects based on pixel-level features^24^. However, these traditional approaches still struggle with capturing the temporal dynamics inherent in surgical procedures, which requires an approach capable of modelling both spatial and temporal data. Automated classification of MIS skills has been described using spatiotemporal motion approaches like HOG and histogram of flow (HOF). Hidden Markov models (HMMs) have been used to represent surgical motion flow^25,26^. Other approaches include linear dynamical systems and bag of features (BOF), which extract features from images to build a visual vocabulary or bag of visual words^27,28^. Support vector machines and random forests are also common machine-learning techniques in MIS^29–31^. With the introduction of Deep Learning (DL), significant improvements have been made in surgical phase identification and instrument detection. The first successful application of DL in MIS was in 2016 with the development of EndoNet, a convolutional neural network (CNN) used for phase detection in laparoscopic surgery^32^. CNNs have proven to be particularly effective in recognizing surgical objects and gestures, although they are typically more suited to still images than video sequences, which require the ability to model temporal dependencies. Researchers in the EndoNet paper augmented a CNN with a HMM technique to improve temporal modelling and identification consistency^33^. Other researchers have since reported the successful use of combining CNNs and HMMs, as well as CNNs and gradient-boosting techniques^34,35^. Gradient boosting is an ensemble technique using decision trees. This led to further research in improving temporal learning in MIS with the addition of long short-term memory (LSTM) neural networks, which allows for more efficiency in identifying phases of surgery and tracking surgical instruments. Combining a CNN and LSTM gives the advantage of more efficiently identifying phases of surgery and tracking surgical instruments^36,37^. The EndoNet researchers published follow-up research comparing a CNN with HMM versus a CNN with LTSM^37^. Jaccard scores for the CNN with LSTM were 0.64, versus 0.62 for the CNN with HMM. Accuracy for the CNN with LSTM was 80.7% versus 71.1% for the CNN with HMM. A significant shortcoming of the HMM is its Markov assumption, which is that the current state depends solely on the previous state. The LSTM, a special type of recurrent neural network (RNN), differs from standard deep neural networks as it has a “memory” function, whereas standard feed-forward deep neural networks work on the assumption that outputs depend solely on their current input, whereas the output of an RNN depends on the sequence of prior information. However, traditional RNN architectures struggle with long-term dependencies, causing an RNN model to have difficulty accurately predicting the current state in longer sequences. LSTMs handle these long-term dependencies by using memory blocks and gates to control the flow of selected useful information to the next cell, discarding irrelevant information^38^.

Some efforts have improved surgical phase identification accuracy using CNNs and LSTMs, with rates between 85-90%^39–42^. These rates are similar to, or slightly better than, inter-rater agreements between expert surgeons when annotating the same images. Deep neural networks have been used for detecting and tracking surgical instruments with an average precision of 91%^43^. However, tool detection and surgical phase have been reported separately in most works using CNNs and LSTMs. The first successful report using a CNN and LSTM to identify surgical phases and instruments in MIS was in 2020 by Jin et al.^44^, who reported precision of 86.9, recall of 88.0, and accuracy of 89.0% when identifying surgical phases, and 89.1 mean average precision for identification of surgical instruments. A recent study by Bamba et al.^45^ used a YOLO V3 CNN which attained good but slightly less impressive results with 80% precision, recall of 92%, and accuracy of 83%. Others reported using a CNN and LSTM architecture to identify instruments and phases of surgery, with a mean average precision of 89.1% and 87.4%, respectively^46^. Some researchers have demonstrated distinguishing between novices and experts using traditional CV methods including background subtraction, thresholding, Hough transform, and straight-line detection for tasks such as suturing, knot tying, needle passing and instrument handling, mostly using simulators or surgical box trainers^47^. One study successfully differentiated experts from novices using performance metrics like task completion time, velocity, work density, and instrument cross-time. Experts consistently outperformed novices across all metrics and showed greater use of their left hand^47^. Other research has focused on systems for detecting and tracking instruments while calculating performance metrics through motion analysis parameters^48^. Although, these systems showed promise in distinguishing novice from expert laparoscopic surgeons, a key limitation was that instrument tips were occasionally obscured by other objects, affecting the accuracy of position data.

**Limitations and key findings:** Despite the reported success of DL in detecting surgical phases and tracking instruments, there is a notable gap in the literature regarding the automated classification of laparoscopic surgical skill levels in simulated settings. There is also a gap in existing datasets to automate this problem. To fill this gap, we introduce the Laparoscopic Surgical Performance Dataset (LSPD), a newly collected dataset specifically designed to classify simulated laparoscopic surgical skill levels. The LSPD dataset addresses the lack of datasets that can classify skill levels in laparoscopic surgery training and represents a significant advancement in the field. Unlike existing datasets, which focus on tracking instruments or recognizing phases of surgery, the LSPD dataset is specifically tailored to evaluate skill performance in laparoscopic surgery at different levels of expertise: novice, trainee, and expert. This dataset is designed for weakly-supervised learning, reducing the reliance on highly detailed frame-level annotations, a major bottleneck in surgical AI development^14,49^. Furthermore, this study explores the application of 3D Convolutional Neural Networks (3DCNNs) for classifying simulated laparoscopic surgical skill levels based on the LSPD dataset. While 3DCNNs have been successfully used in medical imaging to analyze volumetric data such as CT and MRI scans, their application to spatiotemporal feature learning for skill classification in laparoscopic surgery is novel. To our knowledge, this is the first study to apply 3DCNNs to spatiotemporal learning in the context of surgical skill classification, and we argue that this approach has the potential to overcome many of the challenges faced by traditional CV and deep learning methods in this domain. By combining the LSPD dataset with a 3DCNN architecture, we aim to demonstrate a promising new approach to automatically classify performance skill levels based on simulated laparoscopic surgery, with minimal expert annotation. This weakly-supervised approach represents a significant step forward in the development of scalable, automated systems for evaluating surgical skills, which could ultimately streamline the training and assessment of laparoscopic surgeons, and other healthcare professionals.

## Methodology

### Laparoscopic surgical performance dataset (LSPD) -data acquisition

A new dataset was acquired and analyzed to assess simulated laparoscopic surgical performance. The LSPD was created to address a deficiency in the available resources for evaluating surgical skills in laparoscopic simulation training. Unlike existing datasets, which are largely focused on detecting and tracking surgical instruments, identifying anatomical structures, or analyzing surgical phases and robotic gestures, the LSPD is specifically designed to assess the procedural fluency and technical proficiency of surgeons within simulated laparoscopic environments. This specialization makes the LSPD an essential tool for advancing research in surgical skill assessment and simulation-based training programs. The participants for data collection were recruited from local doctor-in-training programs and consultant-level doctors. The study aims to automatically classify performance levels into novice, trainee, and expert groups, and assess the ability of a deep learning model to discriminate skill performance in laparoscopic surgical training skills. Novices are intern doctors with less than 12 months of experience, trainees are those in specialist training programs, and experts are consultant-level surgeons and gynecologists, or senior registrars who have completed their specialist training. All novices, trainees, and experts were recruited voluntarily from university-affiliated teaching hospitals in the Cork City region. Ethical approval was obtained from the Social Ethics Committee (SREC) at University College Cork, and the study followed all guidance and regulations as set out by SREC.

Three laparoscopic surgical simulation training skills were identified using the Laparo$^{\textrm{TM}}$ laparoscopic surgical simulator. Participants watched three short Laparo$^{\textrm{TM}}$ training videos on how to perform the skills before attempting each skill^54^. They could re-watch the videos, ask questions, familiarise themselves with the instruments, or practice a separate skill before attempting any of the three skills. Many researchers have a time limit for attempting a skill, however, this leaves the less experienced at a significant disadvantage, can introduce significant measurement bias, and lastly, time as a metric for performance is controversial^55^. Short videos were collected of participants performing basic laparoscopic surgical simulation skills within the Laparo$^{\textrm{TM}}$ system, from its camera connected to a laptop computer. The three skills varied from relatively easy to very difficult.

**Fig. 2:**
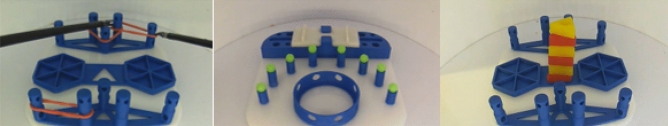
Skills: Bands (left), Stack (center), Tower (right).

The study involved 40 participants, including 8 experts, 12 trainees, and 20 novices. The three tasks were: (1) *bands*, moving elastic bands onto pegs (Fig. 2, left); (2) *stack*, stacking balls on stacks (Fig. 1, center); (3) *tower* aligning rubber triangles into a tower (Fig. 1, right). Videos were collected from each group, with some performing all three skills multiple times. Some videos were discarded due to obstructed views, to reduce bias in the system. The number of videos was, for the expert group: *bands*=4, *stack*=9, and *tower*=6; for the trainee group: *bands*=10, *stack*=11, and *tower*=18; and for the novice group: *bands*=8, *stack*=21, and *tower*=19. This resulted in a total of 106 videos. OpenCV was used to edit the videos to remove noise at the beginning and end while preserving participant actions^56^. The last 60 seconds of each of the video clips in the dataset was used for the analysis due to computational constraints.

Training a deep learning-based video classifier can be challenging due to the need for a large amount of data to avoid over-fitting and to have a generalized model. Data augmentation increases the size and diversity of the training dataset by applying various transformations to the original dataset. It creates new instances of the data with minor alterations while preserving the semantic content of the images^57^. Data augmentation acts as a form of regularisation, preventing over-fitting by introducing variations in the dataset, increasing the model’s robustness and ability to generalize to unseen data. It also minimizes the model’s sensitivity to small changes in input videos^58^. Data augmentation exposes the model to a wider range of data changes, including noise, occlusions, and varying lighting conditions. Hence, it reduces bias in training data by introducing more diverse samples, leading to a more balanced and representative dataset^58^.

For this dataset, the specific forms of data augmentation we applied were: (1) Gaussian blur; (2) adjustments to brightness and contrast; (3) salt and pepper noise; (4) horizontal flipping. Gaussian blur of $\sigma =0.2$ is applied to each pixel using a weighted average, determined by a Gaussian kernel, achieving a slight blur but retaining essential spatial information^59^. The brightness $\alpha$ was adjusted to 1.2 to increase the brightness level in the samples. The contrast was adjusted to $\beta =1.2$ to enhance the difference between light and dark areas of video frames, making edges and features more pronounced resulting in a noticeable but not overly drastic increase in contrast transformation in the samples^60^. In addition, salt and pepper noise was added to about 2% of the pixels in each frame, to prevent excessive distortion of the original data. The final data augmentation technique was horizontal flipping, creating a mirrored version of each frame. This technique doubled again the transformed dataset and made the model invariant to horizontal orientation^61^. The final dataset contained 2244 videos in total.

A weakly supervised approach was taken to discriminating between surgical performance levels using video classification. In this approach, a machine learning model is trained with coarse or noisy labels, rather than fully annotated data. In this case, the labels provided during training do not correspond directly to specific frames or segments of the video but instead indicate general performance levels across entire procedures. General annotation is achieved from the folder-based organization of videos representing different surgical skills and operator levels. This contrasts with fully-supervised learning, where precise labels are available for each frame or action within the video which may be datapoints that pinpoint moments or actions within videos that contributed to the overall performance assessment. Therefore, a weakly-supervised video classifier must infer relevant features from the data, often relying on global cues rather than fine-grained, frame-by-frame information. This might involve recognizing overall procedural fluency, movement smoothness, or the time taken to complete specific steps, rather than detecting individual errors or successes. The absence of detailed annotations can potentially introduce ambiguity between classes, however, there are clear benefits to this approach in significantly reducing expert annotation time and effort.

### Proposed 3DCNN architecture

**Fig. 3:**
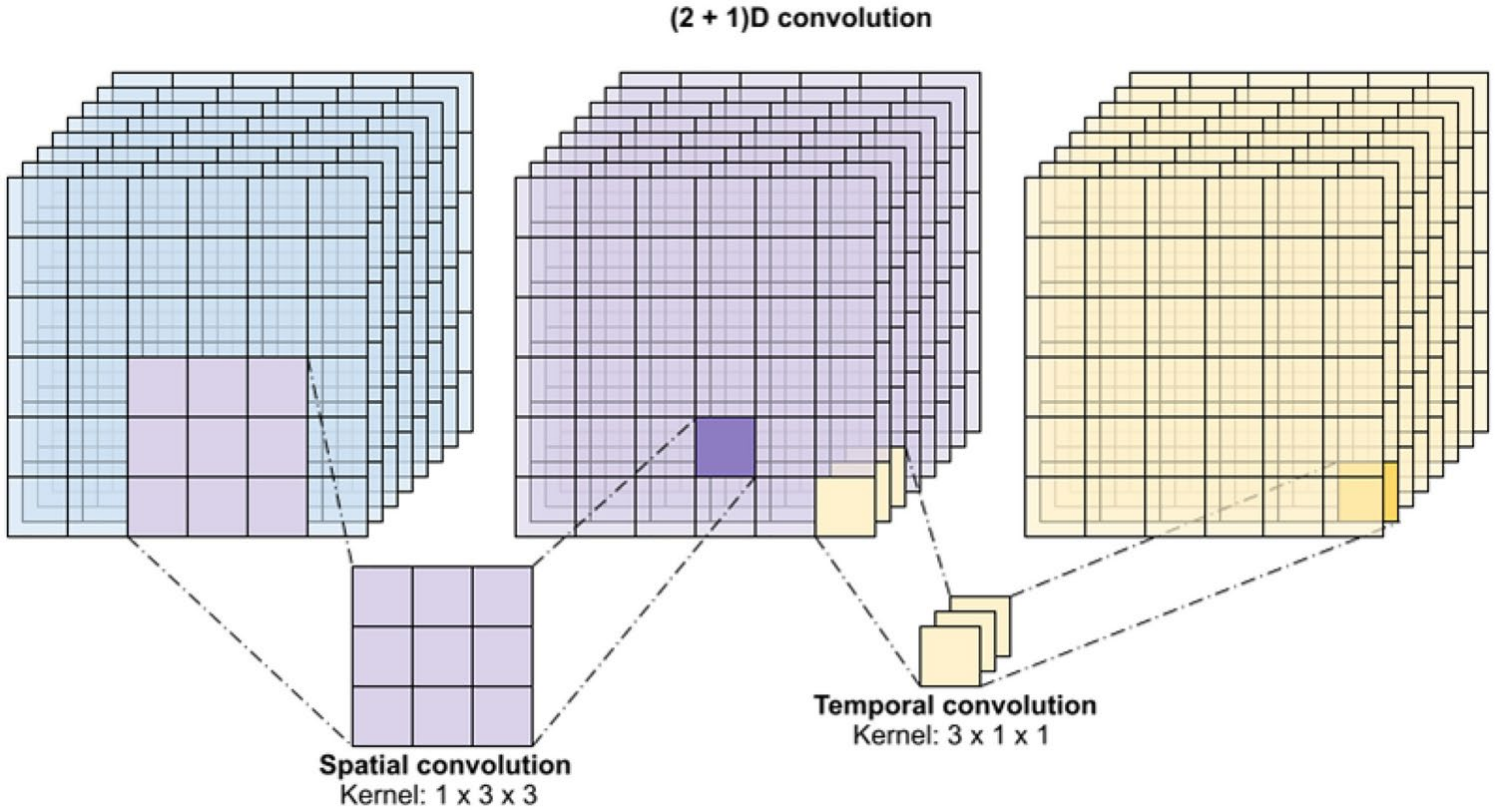
Spatial and temporal kernel sliding in a 3DCNN. [figure from www.keras.io].

Standard 2D-CNNs use a two-dimensional filter to perform convolutions, moving the kernel in two directions. There are also 1D and 3D variants. 1D-CNNs slide kernels along one dimension and are generally used with time-series data. 3DCNNs perform convolutions in three dimensions and are mainly used for video analysis or volumetric data processing, such as MRI or CT scan analysis^62,63^ (Fig. 3). Kernels are small tensors that perform convolution operations on input images, defining a specific pattern or transformation applied to extract features^59^. Each element of the kernel is a weight, which is learned during the training process through back-propagation. They are good at detecting simple features like edges, corners, and textures^63^. Each kernel in a CNN layer is responsible for detecting different aspects of the input data.

**Fig. 4:**
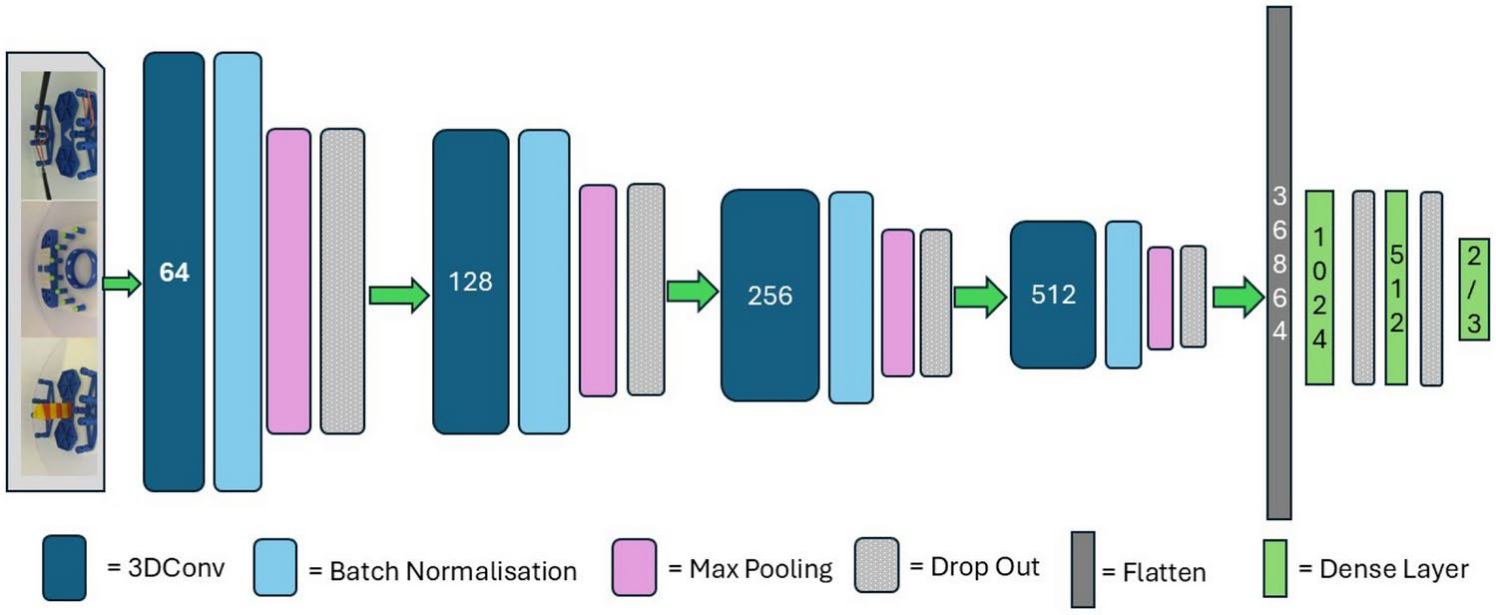
Model architecture.

**Fig. 5:**
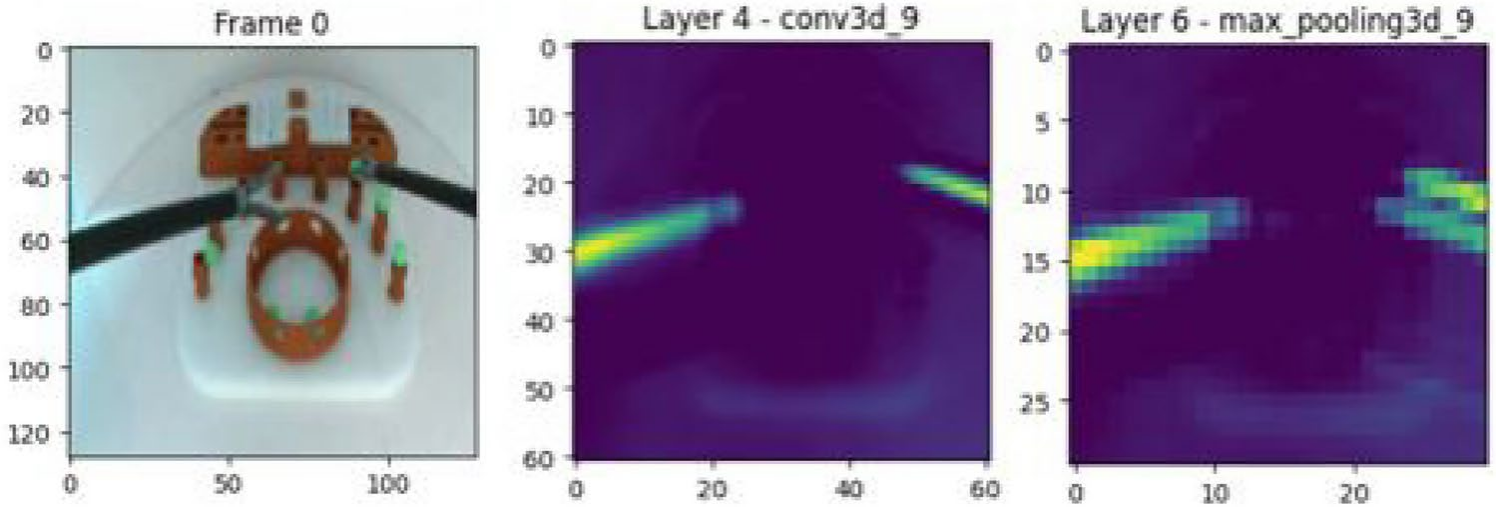
Feature maps, left: input image, centre: conv layer, right: max pooling layer.

The 3DCNN model structure, with four 3D convolution layers (Fig. 4), is optimized for classifying videos. The first, second, third and fourth layer have 64, 128, 256, and 512 kernels of size 3x3x3, respectively. Following each layer, there is a batch normalization, max-pooling, and dropout layer to normalize and reduce the complexity of the model. The final output layer has either one or three neurons depending on whether it is working as a binary classifier or a multi-class classifier. The image size was reduced from its original 1280 x 720 to an input of size 128 x 128 for computational efficiency; however, the essential spatial information was retained at these dimensions (Fig. 5, left). Zero-padding was used to ensure all frames were the same length. Frames were normalized by dividing the pixel values by 255 to bring them within the range [0, 1].

The **ReLU activation function** is used which introduces non-linearity to networks, enabling them to model complex relationships in video frames with diverse patterns and features^64^. It does not suffer from vanishing gradients, which occur when the gradients of activation functions become very small or close to zero. This results in slow or stalled learning in the early layers. ReLU may lead to more efficient learning and generalization through sparse activation, as activated neurons are either fully active when the input is positive or completely inactive when the input is negative^65,66^.

A **batch normalization layer** is introduced after each 3D convolutional layer to improve network training and convergence. This layer normalizes and stabilizes intermediate activations within the network during each training batch, reducing the risk of slow convergence, vanishing, or exploding gradients. Batch normalization standardizes the mean and variance of each feature across a batch, bringing the data closer to a standard Gaussian distribution^66^. It also reduces internal covariate shift, allowing the network to focus on learning higher-level features. Batch normalization can reduce dependence on hyperparameters for performance, such as learning rate, and accelerate model training. Generally, networks trained using batch normalization converge faster than those without. Batch normalization introduces a small amount of noise to the network, similar to dropout or weight decay, which helps prevent the network from relying too heavily on specific activations and encourages learning more robust features. This layer can be effective when used in combination with other regularisation methods^67^.

Following each batch normalization layer is a **max-pooling layer** that uses a 2x2x2 window to slide over the input feature map, which down-sample the spatial dimensions of the feature maps while preserving the most important features (Fig. 5, right). This process reduces computational complexity and improves translation invariance^66–68^.

Following each max-pooling layer, **dropout layers** are introduced which are a regularisation technique that is used to prevent overfitting and improve generalization of the network. The first three dropout layers have small dropout rates of 0.1 and 0.2, while the final two have a rate of 0.5, reflecting approximately half of the neurons in that layer being dropped out in that training iteration. During the inference or prediction phase, no neurons are dropped out, but the weights of the remaining neurons are scaled down by the dropout rate to account for more neurons being active^68^.

The input in the network is then converted into a one-dimensional array through a flatten operation, which reshapes the multidimensional tensor into a one-dimensional vector while maintaining its order. This process is a transition between convolutional layers and **fully connected layers**, as it takes the multi-dimensional tensor as input and outputs a one-dimensional vector to the fully connected layers. The flatten layer in a neural network serves as a transition between these layers, ensuring all elements are laid out sequentially in a single row^66–70^. The first fully connected layer has 1024 neurons, while the second has 512 neurons. The final fully connected layer is the output layer, which consists of three neurons representing each class, expert, trainee, and novice after passing it through the sigmoid activation function.

It uses binary cross entropy as the loss function, optimized with Adam, and accuracy measure as a performance metric. The goal is to predict the correct class label for each input. The output is a probability score for each class, with the binary cross-entropy loss measuring the difference between the predicted probability and true label. The loss function encourages the model to assign high probabilities to the correct class and lower probabilities to the opposite class. The model is penalized when the predicted probability deviates significantly from the ground truth^65^. Gradient descent is used to update the model’s weights and minimize the loss function. This optimization process adjusts the model’s parameters to improve its ability to classify input videos, resulting in higher probabilities for positive videos and lower probabilities for negative ones^72^.

The 3DCNN video classifier underwent cross-validation to reduce overfitting and ensure robust evaluation. The dataset was randomly divided into *k*=5 subset folds, and the model was trained and evaluated *k*=5 times using different folds as validation and training sets. This method provides a more reliable estimate of the model’s performance on different dataset subsets. All experiments were conducted using Google Colab$^{\textrm{TM}}$, a cloud-based Jupyter notebook, and the NVIDIA Tesla$^{\textrm{TM}}$ A100 GPU, Google’s top-performance GPU.

## Experiments & results analysis

### Statistical analysis of videos

Three skills were chosen to discriminate between performance level and were considered at three different difficulty levels ranging from easy to very difficult with *bands* being easy, *stack* moderately difficult and *tower* very difficult. However, statistical analysis of the videos was carried out to quantify this assumption by gauging skill difficulty, and as a benchmark for the model. The assumption was that an easy task would be easy for all performance groups, however it may be difficult for the model to discriminate between performance levels. A very difficult task could be a real discriminator between performance levels, and it would be easier for the model to discriminate between groups if there is less ambiguity between the performance level groups. The scoring system developed for this study was intentionally designed as a basic tool to gauge the relative difficulty of different tasks *(band, stack, and tower)*. The primary objective was to establish a benchmark for task complexity, rather than to evaluate detailed performance outcomes. This tool focuses on end results rather than the nuances of procedural performance, offering a straightforward measure to facilitate comparison across the different tasks.

Time was recorded from the start to the end of each skill, and the median of each group was calculated. A basic scoring system was developed for each skill, with a total score of 8/8 for the *stack* skill. The final score was calculated by the number of balls remaining on the stacks at the end of the skills video. The total performance score for the *band* skill was 6/6, with 1 for each band moved correctly to the correct pegs and 1 for exact symmetry with the starting position pegs. The total score for the *tower* skill was 12/12, with 1 for each of the six triangles placed in the correct location and 1 per triangle for the exactness of direction and angle of points. The median was chosen as the dataset was small with a few outliers and therefore may be a more stable and reliable estimate of the central tendency^65,76^.

**Fig. 6:**
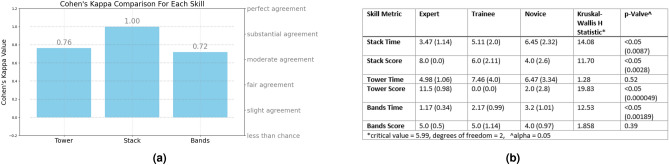
(**a**) Inter-rater reliability of the scoring system for each skill, (**b**) Median time and score for each skill with p-values, SD, and H-statistic.

Two healthcare simulation experts marked 30 videos of skills, with inter-rater reliability measured using Cohen’s Kappa. The *stack* skill had very high agreement (Cohen’s Kappa = 1.0), while the *tower* skill (Cohen’s Kappa = 0.76), and the *band* skill (Cohen’s Kappa = 0.72) had moderately high agreement (Fig. 6a)^66^. As the aim is to measure the performance of the model, rather than human performance, this was considered sufficiently robust for this study.

**Fig. 7:**
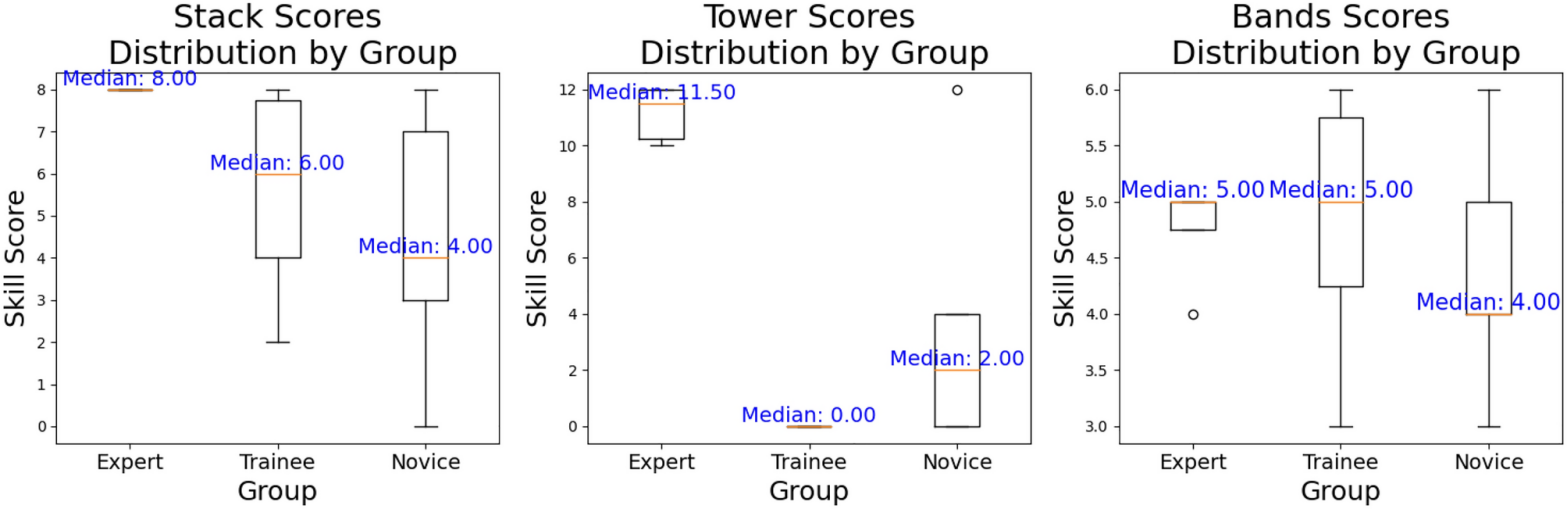
Distribution plots of performance for each skill.

**Fig. 8:**
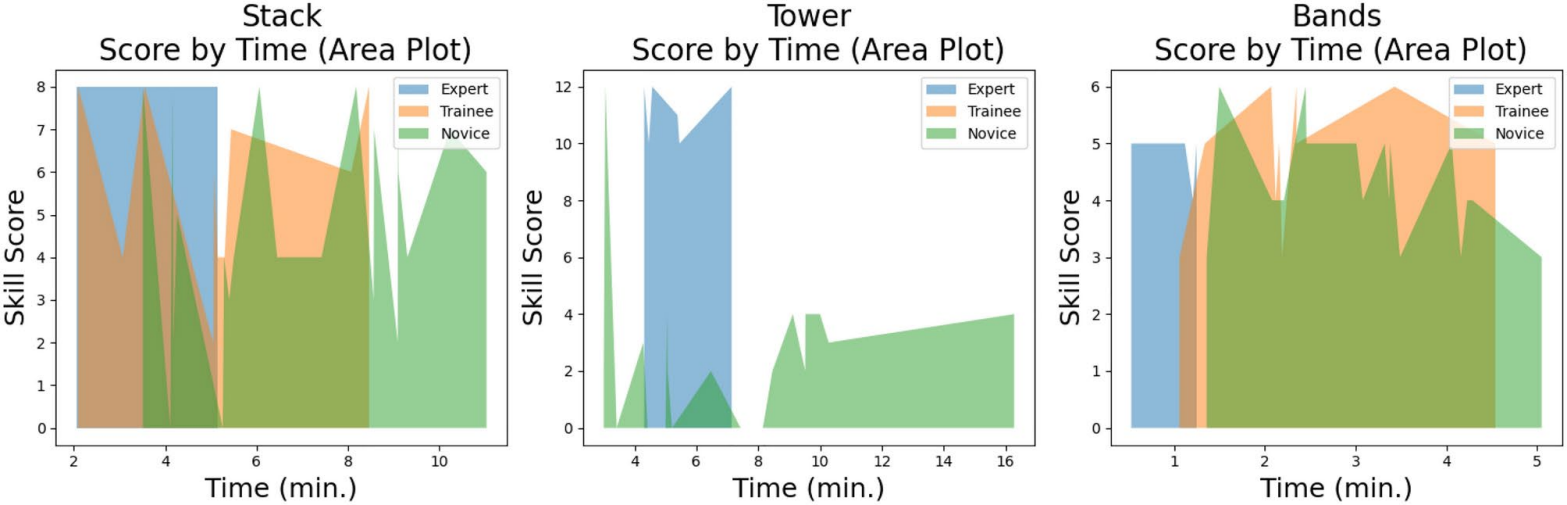
Area plots of time and performance for each skill. *In cases where participants scored full performance scores or zero, the plot collapses to a line.

The median time of experts to complete the *stack* skill was 3.47 minutes, compared to 5.11 minutes for trainees and 5.45 minutes for novices. The median performance score for experts for the *stack* skill was 8/8. This is compared to a median performance score of 6 for trainees and 4 for novices for *stack* skill. The median time of experts to complete the *tower* skill was 4.98 minutes, compared to 7.46 for trainees and 6.47 minutes for novices. The median performance score for experts for the *tower* skill was 11.5/12. This is compared to a median performance score of 0 for trainees and 2 for novices for the *tower* skill. The median time of experts to complete the *band’s* skill was 1.17 minutes, compared to 2.17 minutes for trainees and 3.2 minutes for novices. The median performance score for experts for the *band’s* skill was 5/6 (Fig. 6b). This is compared to a median performance score of 5 for trainees and 4 for novices for the *band’s* skill. See the distribution of performances across skills in Fig. 7 and time plotted against performance for each skill in an area plot in Fig. 8.

### Interpretation of statistical analysis of video results

**Fig. 9:**
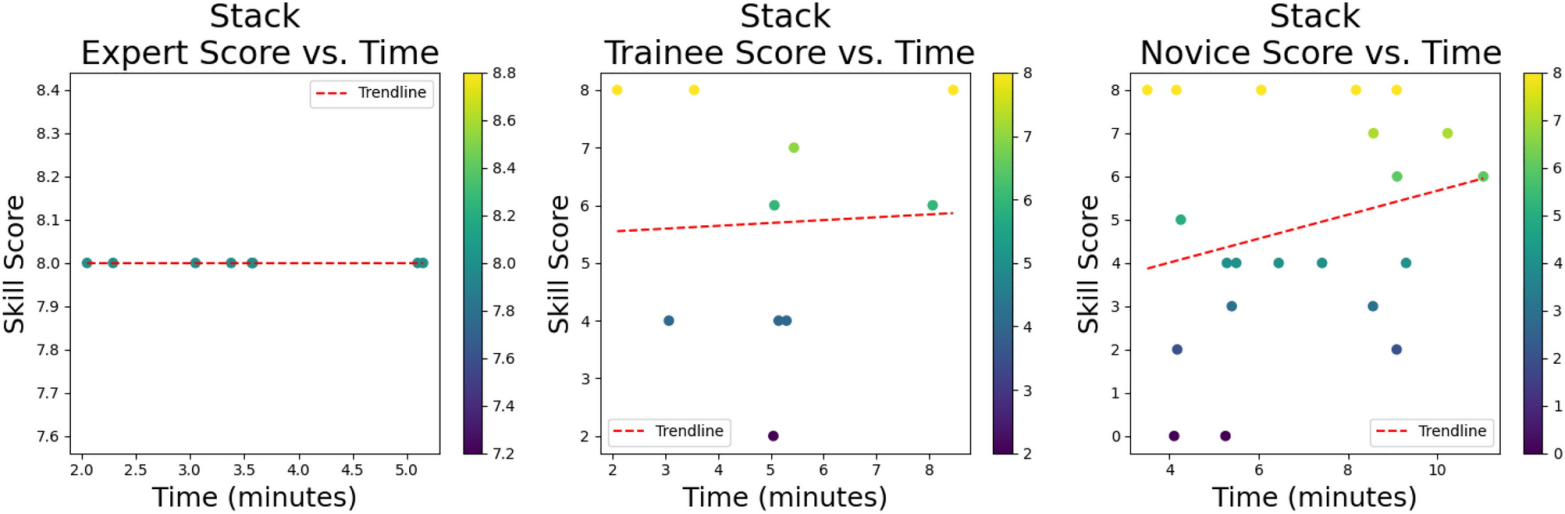
Relationship trend between performance and time: Stack skill.

**Fig. 10:**
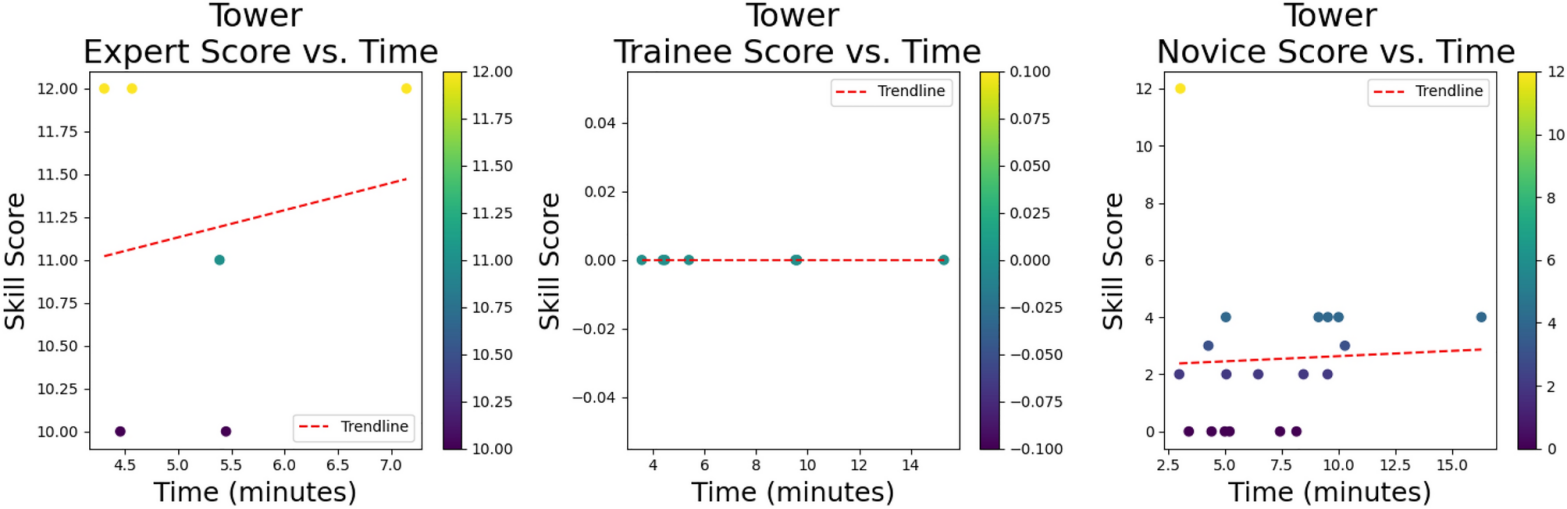
Relationship trend between performance and time: Tower skill.

For the *stack* skill, all experts got a full performance score of 8/8 and finished the skill in a considerably faster time than both the trainees and novices. The trainee’s performance was 6/8 compared to the novice’s performance of 4/8 for this skill and trainees finished the skill marginally faster than the novices, 5.11 minutes versus 5.45. See the relationship trend between performance score and time for each group for the *stack* skill (Fig. 9). The red trend line represents the linear relationship between performance score and time^67,76^. With a median performance score of 11.5 for experts, compared to 0 for trainees and 2 for novices, the *tower* skill was a difficult skill for all groups. Experts completed the skill in 4.98 minutes, whilst trainees took 7.46 minutes to complete it, and novices 6.47 minutes (Fig. 10). Although it would have appeared that novices were speedier and performed better, this was not the case. By the time the video ended, some of the trainees had almost erected the *tower* correctly when they unintentionally toppled it. This skill was also the most challenging, and many novices gave up after an attempt to stack only a few triangles. In fact, the median tower built by the trainee group was 3, over a median time of 6.00 minutes. The range of the size of tower built in this group was zero to five and attempts to build a tower ranged from once to four times. In one instance a trainee built four towers in a 12-minute window of 4 triangles high but knocked it over each time. The rudimentary scoring system simply considers the single tower that is upright at the conclusion of the video, not actual participant performance throughout the entire video.A simple scoring system was selected to provide a consistent and easily interpretable benchmark for assessing task difficulty across different procedural skills. However, we acknowledge that this basic system may not capture the full complexity of procedural execution, particularly for tasks like ’tower’ that require a more nuanced assessment of performance throughout the procedure, where focusing solely on the end result may overlook important aspects of procedural performance.

**Fig. 11:**
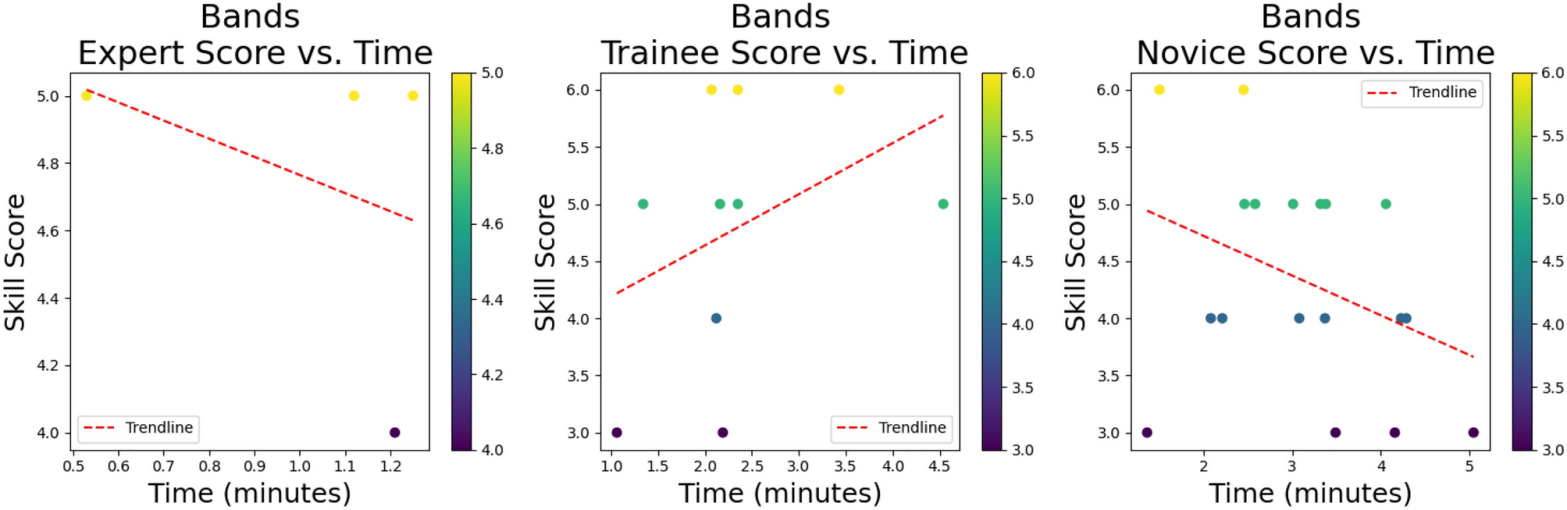
Relationship trend between performance and time: Bands skill.

**Fig. 12:**
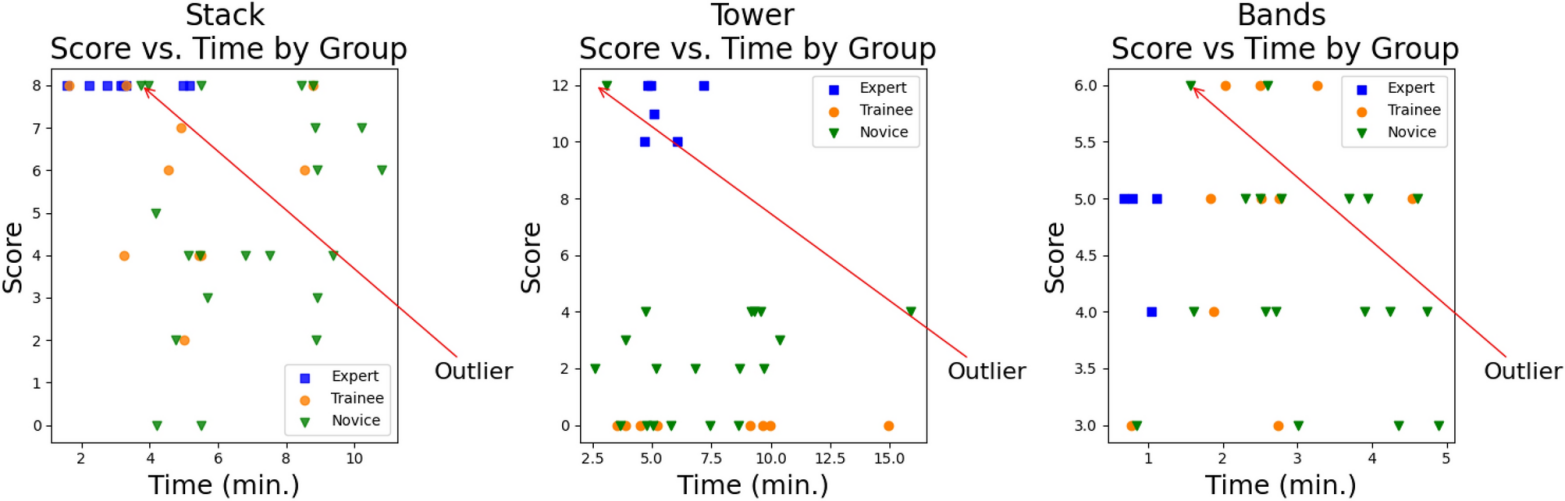
Scatterplots of performance scores vs time for all groups.

For the *band’s* skill, both the experts and trainees received the same performance score of 5 out of 6. However, the experts completed the skill more quickly, in 1.17 minutes as opposed to 2.17 minutes. The novice’s performance was 4, and they took longer to complete it, taking 3.2 minutes (Fig. 11).

It is not surprising that the experts were faster at completing every skill and scored the highest performance score for all three skills. The *tower* skill was the most difficult and a real discriminator between expert and non-expert as reflected in the performance scores. There is the odd outlier within the novice group that completed skills both quickly and with high performance scores, an interesting example of which can be seen at the top left-hand corner of the *tower* skill in Fig. 12. This novice had never undertaken any laparoscopic training, was on a medical rotation, and denied playing a lot of video games. Anecdotally there is a perception of cross-over and correlation between video game playing and laparoscopic skills, however, evidence to support this remains controversial^77,78^.

## 3DCNN classification results

**Fig. 13:**
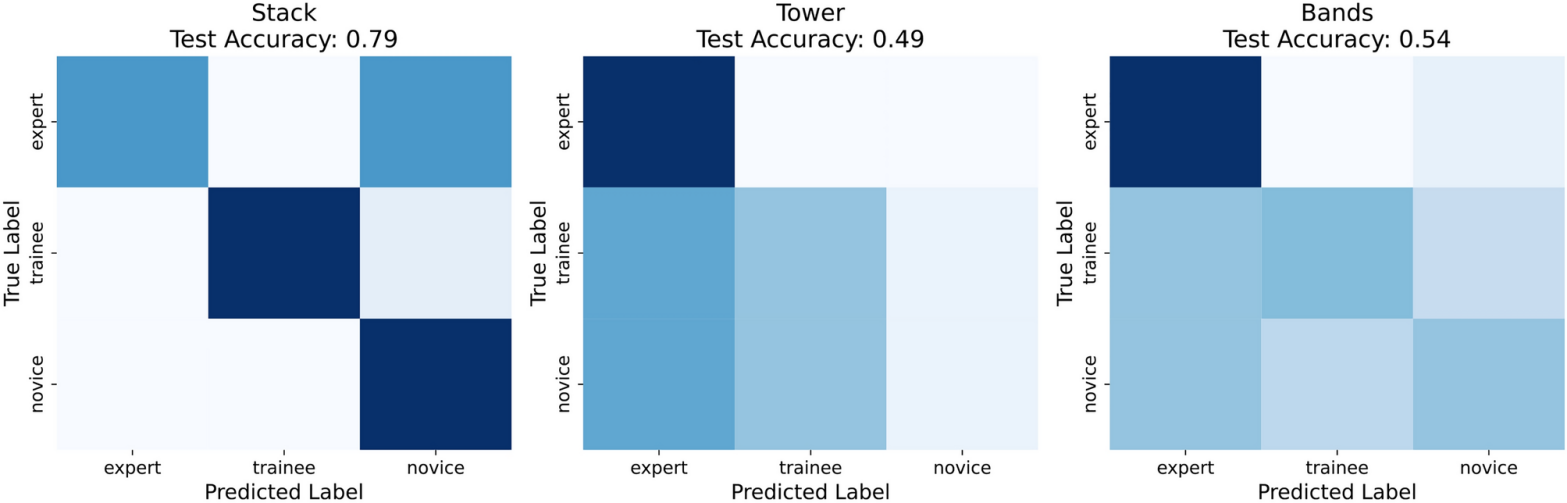
Multi-class confusion matrices.

The proposed 3DCNN model was tested in two ways i.e. as a multiclass classifier and as a binary classifier. Firstly, it is trained to classify among performance classes novice, trainee, and expert over the three skills. However, the model frequently misclassified instances and had poor test accuracy, especially for the *tower* and *bands* skills, as seen in the confusion matrices Fig. 13. The testing accuracy of the *stack* skill (i.e. 79%) is higher than the other two, *tower* (i.e. 49%), and *bands* (i.e. 54%) skills (Table 1). This aligns with the results shown in the statistical analysis i.e. *tower* is the most difficult of the tasks.

**Table 1:** Multiclass vs Binary classifier accuracy.

Multi-class	Accuracy	Binary	Accuracy
Stack	79%	Stack	91%
Tower	49%	Tower	97%
Bands	54%	Bands	79%

**Fig. 14:**
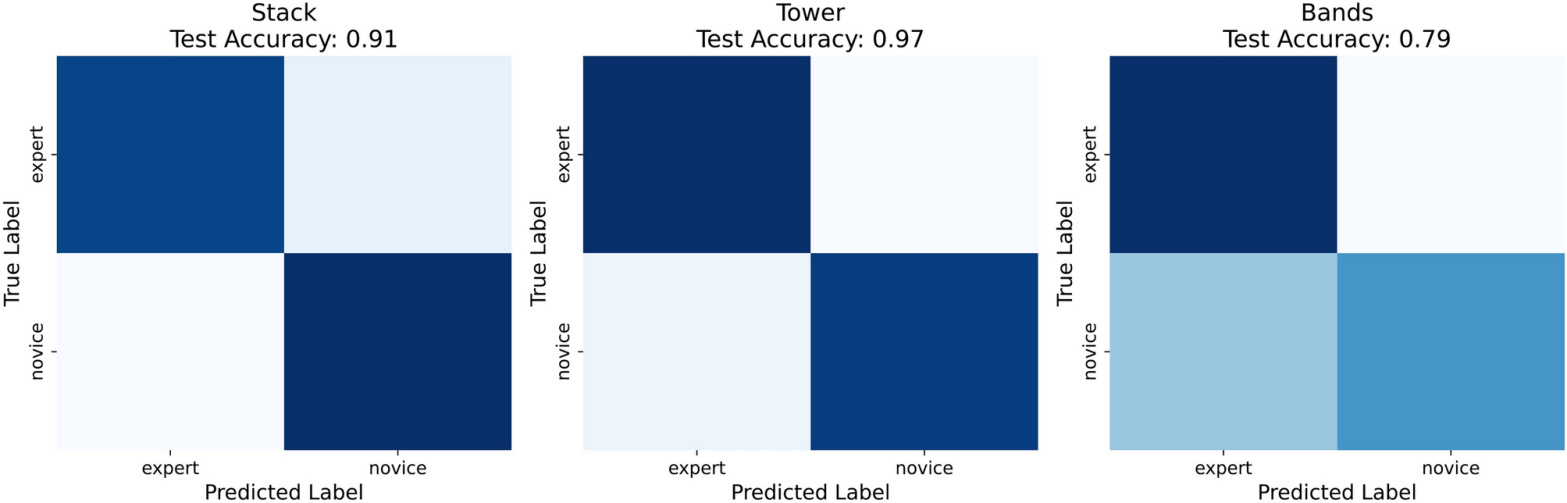
Binary class confusion matrices.

In the second test, the cases classed as trainee were dropped and the model was trained as a binary classifier, with two classes of novice and expert for the same three skills, with much-improved performance as shown in Fig. 14. Being able to reliably and accurately classify expert proficiency from novices was seen as most important. All three skills were trained with a cross-validation procedure (*k*=5). The data was divided into training and testing i.e. 80% for training and the remaining 20% for testing. From the training data, 20% was used for validation. The data was shuffled for randomness.

**Table 2:** Precision, Recall, F1 Score and AUC for each skill for Binary classifier.

	Precision	Recall	F1 Score	AUC
		Stack		
Expert	0.94	0.88	0.91	0.92
Novice	0.88	0.94	0.91	0.89
		Tower		
Expert	0.95	1.0	0.97	0.99
Novice	1.0	0.95	0.97	0.99
		Bands		
Expert	0.71	0.97	0.82	0.86
Novice	0.96	0.61	0.74	0.79

The test accuracy of the stack skill was 91%. Expert class precision was 0.94 and recall was 0.88. Novice class precision was 0.91. Both classes had an F1 score of 0.91. The expert class had an AUC of 0.92 and the novice class had 0.92. The tower skill’s test accuracy was 0.97. Expert class precision was 0.95 and recall 1.0. Novice class precision was 1.0, and recall was 0.95. Both classes had an F1 score of 0.97. The AUC for expert and novice classes was 0.99. The model has excellent predictive ability and can discriminate easily between these class instances for both the stack and tower skills. The band skill’s test accuracy was 79%. Expert class precision was 0.71, and recall of 0.97. The expert class achieved an F1 score of 0.82. Novice class precision was 0.96, and the recall score was 0.61. The novice class has an F1 score of 0.74. The model’s AUC for the expert class was 0.86 and 0.79 for the Novice class (Table 2). The model achieved reasonable performance for this skill however, it struggled to correctly classify all experts as experts and missed significant numbers of novices within the dataset. This is not unsurprising as it was the easiest skill according to the statistical analysis.

### Human performance and results

The study involved human raters with healthcare simulation expertise classifying skill performance levels into three groups (expert, trainee, and novice) and two groups (expert and novice) on separate datasets. The goal was to gauge human-level performance and compare it with the model’s performance. Two datasets were randomly selected with balanced numbers of each class within each dataset, with 63 videos for the three groups and 60 videos for the two groups. The raters watched Laparo$^{\textrm{TM}}$ training videos for each skill and were presented with examples of real performance from each group. For the first task, the raters were asked to classify for each of the three skills whether they believed the participant was an expert, trainee, or novice. For the second task, the raters were asked whether they believed the participant was an expert or a novice. The two raters were independent of each other, and their predictions were measured from the ground truth labels. In both instances, raters used the basic scoring metrics described in the statistical analysis section.

#### Human performance with three classes (multi-class)

**Fig. 15:**
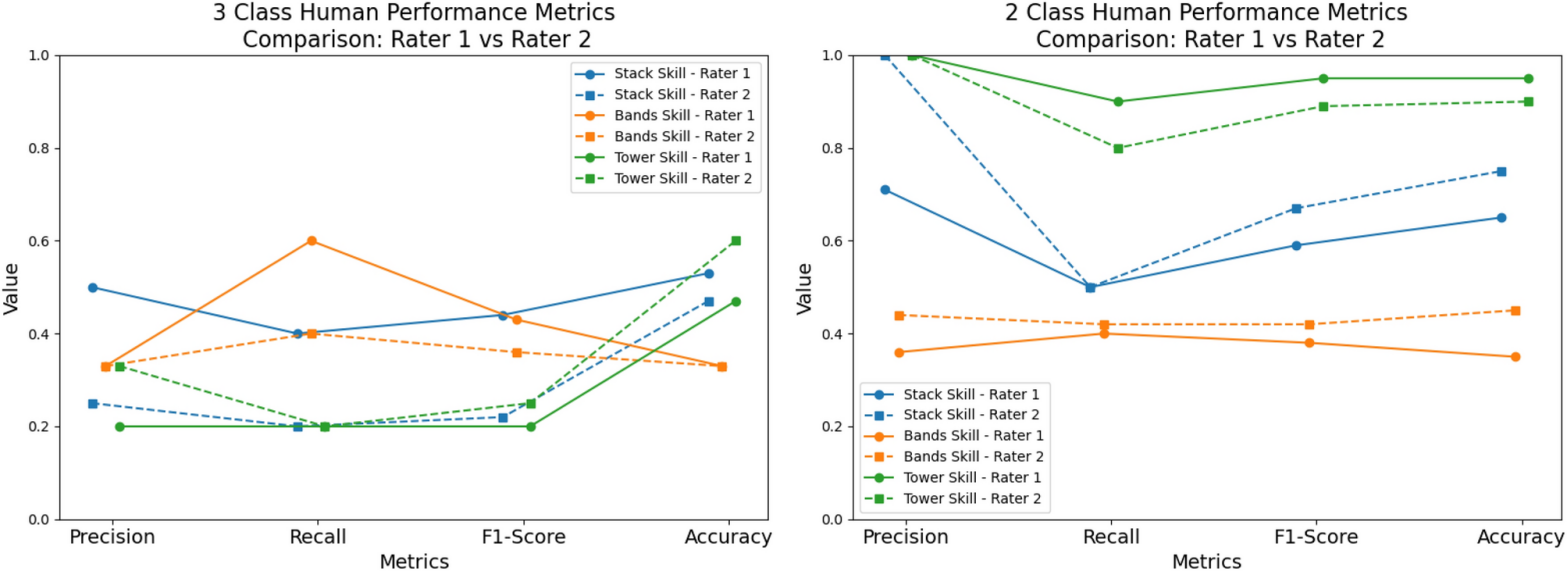
Human performance metrics. Left: 3 classes, Right: 2 classes.

**Fig. 16:**
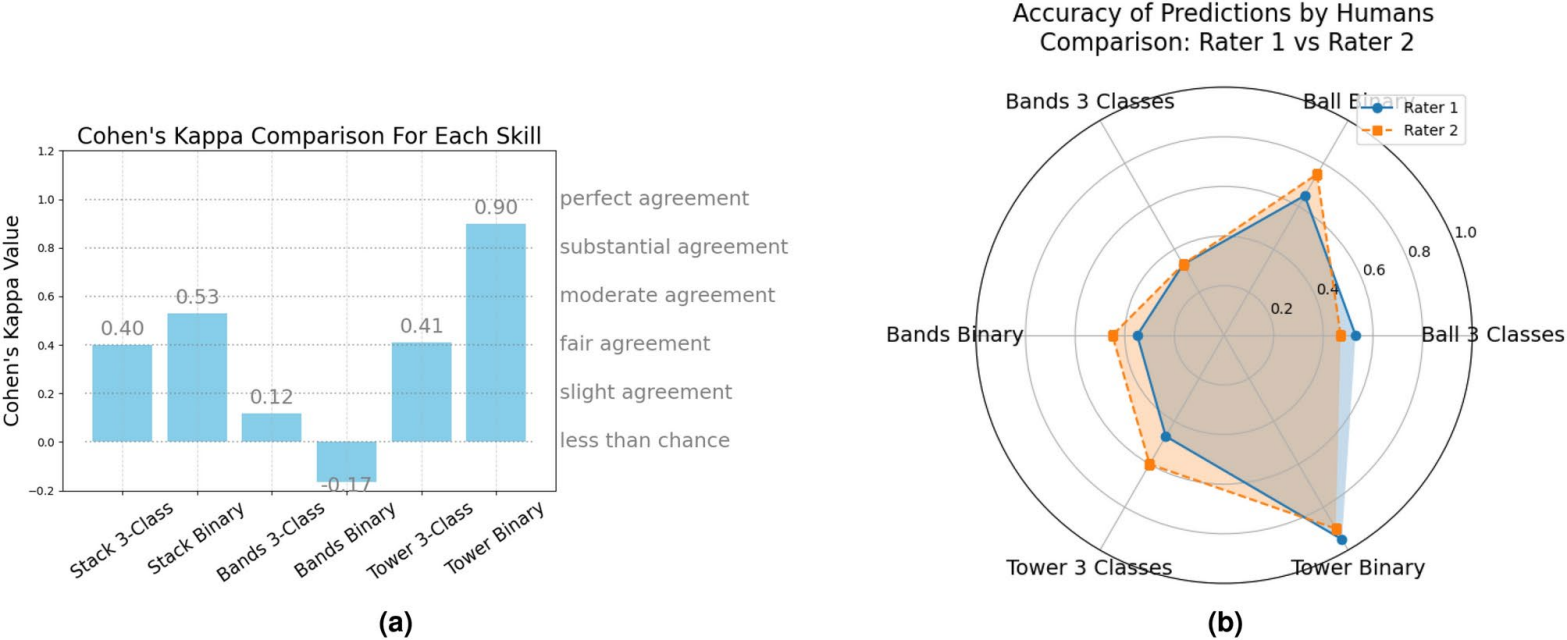
(**a**) Cohens Kappa for each skill for the three-class problem and two-class problem, (**b**) Accuracy of all predictions between both raters.

With three classes for the stack skill, Rater-1 had an accuracy of 53%, precision of 0.5, recall of 0.4, and F1 score of 0.44. Therefore, rater-1 predicted about 50% of the correct classes, reflecting the ratio of true positive predictions to the total number of predictions made for each class. Rater-1 captured about 40% of the actual instances of each class, or the ratio of true positive predictions to the total actual instances of each class. Poor performance is reflected in the low F1 score^64^. Rater-2 had an accuracy of 47%, precision of 0.25, recall of 0.2, and F1 score of 0.22 when classifying between three classes for the *stack* skill (Fig. 15, left). Cohen’s Kappa coefficient was used to measure agreement between the two raters, beyond what would be expected by chance. It is particularly useful in measuring inter-rater reliability and agreement between categorical data^65^. Cohen’s Kappa ranges between -1 and 1, where -1 indicates complete disagreement and 1 indicates perfect agreement. The Cohen’s Kappa between Rater-1 and Rater-2 for the *stack* skill is 0.4, which is considered fair agreement (Fig. 16a). For the *tower* skill, Rater-1 had an accuracy of 47%, precision of 0.2, recall of 0.2, and F1 score of 0.2. Rater-2 had an accuracy of 60%, precision of 0.2, recall of 0.33, and F1 score of 0.25 (Fig. 15, left). The Cohen’s Kappa between the two raters for the *tower* skill was 0.41, which is also considered fair agreement (Fig. 16a). For the *band’s* skill, Rater-1 had an accuracy of 33%, precision of 0.33, recall of 0.6, and F1-score of 0.43. Rater 2 also had an accuracy of 33% for this skill. Rater-2’s precision was 0.33, the recall was 0.4, and the F1 score was 0.36 (Fig. 15, left). The Cohen’s Kappa between Rater-1 and Rater-2 for the *band’s* skill was 0.12, which is considered very low (Fig. 16a).

#### Human performance with two classes (binary)

When performing a binary classification between expert and novice on the *stack* skill, Rater-1 had an accuracy of 65%, precision of 0.71, recall of 0.5, and F1-score of 0.59. Rater-2 had an accuracy of 75%, precision of 1.0, recall of 0.5, and F1 score of 0.65 (Fig. 15, right). The Cohen’s Kappa between the two raters for the binary classification of the *stack* skill was 0.53, which is considered between fair and moderate agreement (Fig. 16a). For the *tower* skill, Rater-1 had an accuracy of 95%, precision of 1.0, recall of 0.9, and F1 score of 0.95. Rater-2 had an accuracy of 90%, precision of 1.0, recall of 0.8, and F1 score of 0.89 (Fig. 15, right). The Cohen’s Kappa between the two raters was 0.9, indicating substantial agreement (Fig. 16a). For the *band’s* skill, Rater-1 had an accuracy of 35%, precision of 0.36, recall of 0.4, and F1 score of 0.38. Rater-2 had an accuracy of 45%, precision of 0.44, recall of 0.4, and F1 score of 0.42 (Fig. 15, right). The Cohen’s Kappa between the two raters for the *band’s* skill was -0.18, which is less than chance (Fig. 16a). In other words, this means that the coefficient indicates that the observed agreement between the two raters is worse than would be expected by random chance.

### Human performance with two classes (binary)

#### Human performance discussion

Both Rater-1 and Rater-2 had more difficulty classifying 3 groups, rather than two groups. The accuracy of predictions between both Rater-1 and Rater-2 are relatively similar (Fig. 16b). With regards to the *stack* and *tower* skills, both raters could make predictions above random chance when classifying between three classes, however, accuracy was still not particularly useful between 47%-60%. Both raters had an accuracy of 33%, which is no better than random for the *band’s* skill when attempting to classify between three groups. When the trainee class was removed, accuracy improved considerably for the *tower* skill to 90% and above, and moderately for the *stack* skill to between 65%-75%. However, there was only a slight increase in the *band’s* skill to 35-45%. The poor Cohen’s Kappa between the raters with this skill indicates, that there was significant disagreement between the classifications. Indicating that the band’s skill is particularly difficult to classify between skill levels, both as a multi-class problem and as a binary problem for humans. There was a significant increase in the accuracy levels in all skills except the *band’s* skill when the trainee group was removed (Fig. 16b). This indicates that the trainee class was possibly problematic for humans to predict in the presence of expert and novice classes.

## Conclusion

This study successfully demonstrated automated assessment of laparoscopic surgical performance in a simulated setting using a 3DCNN model on a custom dataset and lays the foundation for a new research area. The model demonstrated excellent performance and predictive ability for both the *tower* (accuracy 97%) and *stack* (accuracy 91%) skills, and reasonable performance and predictive ability for the *band’s* skill (accuracy 79%) on test data, as a binary classifier. This approach is viable for a binary classification to discriminate between expert and novice classes performing basic simulated laparoscopic surgical skills in a desktop laparoscopic surgical simulator. However, this approach could be expanded to other skills beyond simulated laparoscopic skills and has scope for assessment of performance for other procedural and clinical skills^79,80^. There is significantly less domain expert time needed using this approach as there is no need for frame-level annotation, which is well recognized as a significant bottleneck and impedance to the development of surgical, and clinical simulation-based intelligent assessment tools in general^9^.

The model encountered difficulties in predicting three distinct performance skill levels in a multi-class classification problem. Similarly, human raters also struggled to accurately classify performance skill levels in this context. It is not surprising that the model showed the lowest accuracy when predicting the band’s skill, as statistical analysis of the videos revealed no significant difference in mean performance for this skill. Human raters also had difficulty distinguishing skill levels in this area, whether approached as a multi-class or binary classification problem. When comparing the model’s performance to that of human raters, similar trends were observed: human accuracy was notably higher for binary classification tasks (ranging from 65% to 75%) than for multi-class tasks (ranging from 47% to 53%). While this comparison offers some insight, a larger sample of human raters would be needed to draw more definitive conclusions.

The distribution of skill levels in the trainee group was too broad, with some participants near novice and others near expert, which made it especially challenging for the model to accurately classify the group (Fig. 13). Human raters also found this difficult. Future work would benefit from more detailed definitions of each skill level, particularly an intermediate group such as trainees. A clearer definition of what constitutes a trainee should be established at the outset. The dataset was also small, comprising 106 original videos, with data augmentation accounting for the remainder of the dataset. Ideally, the original dataset would be larger to provide greater diversity, which may make it easier for the model to differentiate between the classes.

A 3DCNN automatically learns and processes spatiotemporal features, which are crucial in tasks like laparoscopic skill classification, where the movement and coordination of instruments over time are key to determining skill level. This research demonstrated that a 3DCNN model can classify skill levels efficiently and automatically from video data, significantly speeding up the process of skill evaluation, allowing for faster assessments in surgical training programs, with the potential of making the model highly valuable for further development in real-time or large-scale applications. Fast and efficient automated identification of non-experts allows for faster throughput through training programmes and allows for more timely expert feedback and intervention, with more focused and deliberate practice. Furthermore, as our model could robustly discriminate between performance skills levels, it offers potential towards standardising non-subjective approaches to automating skills assessment in laparoscopic surgery and other healthcare domains.

We have demonstrated that weakly-supervised methods using a 3DCNN is a viable approach to automatically discriminate between performance skills in simulated laparoscopic surgical skills using the LSPD dataset. The videos were relatively short due to high computational demands, future work could look at augmented approaches of attention mechanisms, or temporal segmentation to increase the video length sequences and widen the application of use of this approach within the healthcare simulation field.

## Data Availability

The data that support the findings of this study are available on request from the corresponding author, [DP]. The data are not publicly available due to containing information that could compromise the privacy of research participants.
